# Prediction of the Number of Patients Infected with COVID-19 Based on Rolling Grey Verhulst Models

**DOI:** 10.3390/ijerph17124582

**Published:** 2020-06-25

**Authors:** Yu-Feng Zhao, Ming-Huan Shou, Zheng-Xin Wang

**Affiliations:** School of Economics, Zhejiang University of Finance & Economics, Hangzhou 310018, China; yfzhao@zufe.edu.cn (Y.-F.Z.); zxwang@zufe.edu.cn (Z.-X.W.)

**Keywords:** COVID-19, grey prediction, rolling mechanism, grey Verhulst model

## Abstract

The outbreak of a novel coronavirus (SARS-CoV-2) has caused a large number of residents in China to be infected with a highly contagious pneumonia recently. Despite active control measures taken by the Chinese government, the number of infected patients is still increasing day by day. At present, the changing trend of the epidemic is attracting the attention of everyone. Based on data from 21 January to 20 February 2020, six rolling grey Verhulst models were built using 7-, 8- and 9-day data sequences to predict the daily growth trend of the number of patients confirmed with COVID-19 infection in China. The results show that these six models consistently predict the S-shaped change characteristics of the cumulative number of confirmed patients, and the daily growth decreased day by day after 4 February. The predicted results obtained by different models are very approximate, with very high prediction accuracy. In the training stage, the maximum and minimum mean absolute percentage errors (MAPEs) are 4.74% and 1.80%, respectively; in the testing stage, the maximum and minimum MAPEs are 4.72% and 1.65%, respectively. This indicates that the predicted results show high robustness. If the number of clinically diagnosed cases in Wuhan City, Hubei Province, China, where COVID-19 was first detected, is not counted from 12 February, the cumulative number of confirmed COVID-19 cases in China will reach a maximum of 60,364–61,327 during 17–22 March; otherwise, the cumulative number of confirmed cases in China will be 78,817–79,780.

## 1. Introduction

**Figure 1:**
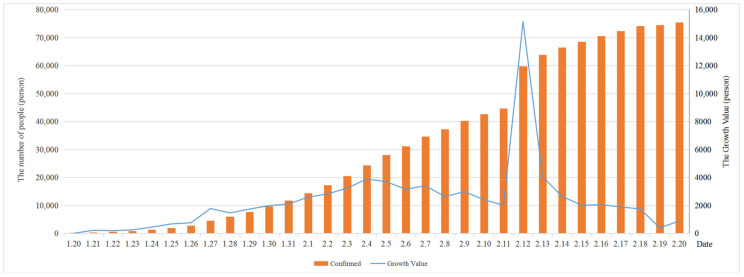
Changes in the numbers of confirmed and newly confirmed cases in China from 20 January to 20 February.

**Figure 2:**
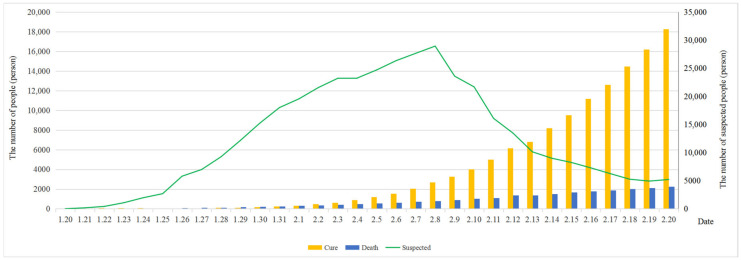
Cumulative numbers of suspected cases, deaths and cured patients in China from January 20 to 20 February.

An epidemic of the novel coronavirus disease 2019 (COVID-19) broke out in Wuhan City, Hubei Province, China, in early 2020, and spread rapidly in China and across the world, causing tens of thousands of people to be infected with the virus. On 21 January, the prevention and control of the epidemic sprouted at the national level, and many provinces in China launched the first-level emergency response of epidemic prevention. By 30 January, more than 7000 COVID-19 infection cases had been confirmed in China and more than 50 cases had been confirmed in countries and regions outside China, so the World Health Organization (WHO) had declared the outbreak of the novel coronavirus in China as a public health emergency of international concern (PHEIC). According to the latest data (http://www.nhc.gov.cn/) released by the National Health Commission of the People’s Republic of China, it can be seen from Figure 1 that the number of confirmed cases increases in an S-shaped trend. Although the cumulative number is rising, the number of newly confirmed cases reached an inflection point, reaching the peak on 4 February and then gradually decreasing. The number of newly confirmed cases rose excessively on 27 January due to the increase in the number of institutions with the capacity of pneumonia detection. Figure 2 demonstrates that the number of suspected cases rose until 8 February and then decreased, while the number of deaths slowly increased and reached 2239 in total on 20 February. Moreover, the number of cured patients significantly increased and reached 18,278 on the same day. On 11 February, the new coronavirus disease was officially named coronavirus disease 2019 (COVID-19) by the WHO. The International Committee on Taxonomy of Viruses (ICTV) named the virus strain as severe acute respiratory syndrome coronavirus 2 (SARS-CoV-2).

The accurate prediction of the number of patients infected with COVID-19 in China is undoubtedly of great significance for implementing prevention and control measures and carrying out economic and social activities. Considering the limited sample size since the outbreak of the epidemic and that a large sample size is needed to use the classical statistical prediction method, the present research used the grey system theory, which can be modeled with only four data points [1,2]. On this basis, a rolling grey Verhulst model and its derived models were established to predict the change trend of the number of cases of COVID-19 infection in China.

## 2. Literature Review

Since the outbreak of the epidemic of COVID-19, many scientists in the world have studied the causes and mechanisms of spread of the virus, along with relevant treatment programs. For example, based on the empirical analysis of a large amount of genomic data on a global scale, Chen et al. [3] firstly tried to explain the reasons for rapid mutation, multiple hosts and strong host adaptability of *Betacoronavirus* at the molecular level. Xu et al. [4] found genetic evolutionary relationships of the novel coronavirus with the severe acute respiratory syndrome (SARS) coronavirus and the Middle East respiratory syndrome (MERS) coronavirus. Furthermore, Lu et al. [5] found that this virus has specific nucleic acid sequences that are different from those of the known coronaviruses. Zhang et al. [6] studied and summarized the new methods for detecting common respiratory viruses. Koo et al. [7] found that a variety of interventions to maintain body distance are effective in reducing the number of SARS-CoV-2 cases. Bi et al. [8] tracked COVID-19-infected persons and their close contacts, showing that detection, isolation and tracking of cases can reduce the spread of the virus and help control the epidemic. Archer et al. [9] used patients from 24 countries/regions, including Europe and the United States, as data sources to investigate, for the first time, the impact of SARS-CoV-2 infection on pulmonary complications and mortality.

To predict the number of people infected with the SARS-CoV-2 accurately, scholars have established a series of models, generally divided into three categories: the basic infectious disease models in the fields of mathematics and medicine [10,11,12,13], the economic models based on traditional statistical methods [14,15,16] and the algorithm models based on machine learning [17,18,19]. However, the above prediction models have some flaws. For example, the assumptions of the infectious disease model are strong, assuming that there is no super communicator, and are easily affected by factors such as geography; economic models or intelligent algorithms have strict requirements on data, requiring a large amount of data to be trained and tested to obtain relatively accurate prediction results. In response to such new viruses, with low data availability and incomplete cognition, the above models may not be applicable. For this reason, this research selected an innovative topic as the research object, namely the prediction of the final number of infected cases, which can provide a scientific basis for the government policies.

Grey system theory and grey prediction models have been widely used in many fields, such as economics [20], demand prediction [21,22] and environmental protection [23,24], since being proposed. The traditional GM(1,1) and the grey Verhulst model are the core grey prediction models. Bao et al. [25] predicted all factors of disability in middle-aged and old people and the probability of specific injuries through the grey GM(1,1) model and found that non-communicable diseases (NCDs) are still the main threats to health for the elderly. In order to accurately predict the development of human echinococcosis in Xinjiang Uygur Autonomous Region, China, Zhang et al. [26] made short-term predictions using three models, i.e., a traditional GM(1,1) model, a grey-periodic extensional combinatorial model (PECGM(1,1)) and a flamelet-generated manifold (FGM(1,1)) model optimized by Fourier series. In the meanwhile, based on the transmission mechanism of echinococcosis, they established a prediction model for dynamic epidemics that can effectively predict the future development trend of epidemics. The traditional GM(1,1) model is mainly applicable to the sequences with strong exponential laws and can only describe the monotonic change process. However, the grey Verhulst model has strong prediction capability for the non-monotonic swinging developmental sequences or the saturated S-shaped sequences due to the first-order accumulated generating operation (AGO-1) of the original data. In recent years, some scholars have explored the optimization of initial conditions [27] and background values [28,29], the research of model properties [30,31] and the accuracy improvement [32] of the grey Verhulst model.

For application scenarios of the grey Verhulst model, some scholars have conducted research based on real socioeconomic systems, which to some extent reflects the effectiveness and superiority of the grey Verhulst model in comparison with the traditional model. In order to improve the accurate prediction of short-term traffic speed and travel time, Bezuglov and Comert [33] utilized the GM(1,1) model, the GM(1,1) model modified by Fourier error and the grey Verhulst model modified by Fourier error for prediction. The results demonstrate that the grey Verhulst model modified by Fourier error can better process sudden changes in the parameters of a traffic system sequence. Wang and Li [34] constructed a non-equal-interval grey Verhulst model and its derived model and optimized parameters of the models by using particle swarm optimization. On this basis, they verified the environmental Kuznets curve (EKC) of carbon dioxide emission in China by discussing the relationship between carbon dioxide emission and economic growth using a grey model. By building a grey Verhulst model, Wu et al. [35] predicted comprehensive air quality indexes in the Chinese cities of Beijing, Tianjin and Shijiazhuang, and the results show that the input of the government can promote improvement of air quality to some extent. Zhang et al. [36] combined the Verhulst model with the BP neural network to gain complementary advantages and improve prediction accuracy and stability. By utilizing the grey Verhulst model, Wang et al. [37] predicted the state of the iron and steel industry in 2025 based on the relationship between carbon emission of the industry and economic growth from 2001 to 2016; they also put forward relevant policy implications. In order to increase the predictive ability of the initial model, the model is changed appropriately to better adapt to the current needs. By introducing a new non-homogeneous exponential function, Zeng et al. [38] constructed a grey N-Verhulst model that overcame the defects of parameter dislocation and unreasonable initial value selection of the traditional Verhulst model. In addition, some scholars have built grey models based on a rolling mechanism to explore the hidden useful information of the original data sequence and improve the modeling accuracy. Akay and Atak [39] proposed a grey prediction model based on a rolling mechanism to predict the total and industrial power consumption in Turkey. The results demonstrate that the grey model based on a rolling mechanism shows greatly improved prediction accuracy. Considering the complex randomness and nonlinearity of short-term traffic flow, Xiao et al. [40] proposed a seasonal grey rolling prediction model based on the cycle truncation accumulated generation method and a rolling mechanism. Xu et al. [41] established a BR-AGM(1,1) model based on the adaptive rolling mechanism to predict greenhouse gas emissions in China and discussed the policy significance of model overfitting and the modeling process. Şahin [42] used a metabolism grey model, a nonlinear metabolism grey model and the optimized versions of the two to predict greenhouse gas emissions in Turkey. The results demonstrate that prediction accuracy of the optimized nonlinear metabolism model based on a rolling mechanism is higher.

The reminder of the research is arranged as follows: Section 3 introduces the traditional grey Verhulst model, the derived model of the grey Verhulst model, the grey Verhulst model based on a rolling mechanism and its derived models; Section 4 shows the prediction and empirical results of pneumonia infection in China; and the conclusions are made in Section 5.

## 3. Models and Methods

The grey Verhulst model is an effective model to describe and predict a process with a saturation state (S-type) under the condition of small samples; it is commonly used in prediction of population, biological reproduction and product life. Under strict anti-epizootic measures in China, it is assumed that COVID-19 cannot spread indefinitely in China and the cumulative number of confirmed cases will not increase indefinitely and will eventually converge to the corresponding saturation value. Therefore, the grey Verhulst model is suitable for modeling growth and changes in the number of virus infection cases, especially for prediction of the final value and inflection point of the number of confirmed cases. The grey Verhulst model selected in this study is proved to be able to predict non-linear data changes with small errors in multiple case studies [43]. Furthermore, the rolling grey Verhulst model established based on a rolling mechanism and its derived model can capture the dynamic characteristics of the future development trend of the system. To be clear, the model in this paper is only applicable to data with S-shaped growth, while the mortality rate and the number of deaths largely depend on the influence of a country’s medical system, population parameters and various disease characteristics, which do not conform to the assumptions of the model and may lead to a large error.

### 3.1. A Brief Introduction to the Grey Verhulst Model

**Definition** **1.** *It is assumed that the original sequence is $X^{\left(0\right)}=\left(x^{\left(0\right)}\left(1\right),x^{\left(0\right)}\left(2\right),\cdots ,x^{\left(0\right)}\left(n\right)\right)$, while 1-AGO sequence of $X^{\left(0\right)}$ is $X^{\left(1\right)}=\left(x^{\left(1\right)}\left(1\right),x^{\left(1\right)}\left(2\right),\cdots ,x^{\left(1\right)}\left(n\right)\right)$, in which $x^{\left(1\right)}\left(k\right)=\sum_{i=1}^{k}x^{\left(0\right)}\left(i\right)$(k=1,2,⋯,n). $Z^{\left(1\right)}$ represents the generated mean sequence of consecutive neighbors of $X^{\left(1\right)}$, namely $Z^{\left(1\right)}=\left(z^{\left(1\right)}\left(2\right),z^{\left(1\right)}\left(3\right),\cdots ,z^{\left(1\right)}\left(n\right)\right)$, in which $z^{\left(1\right)}\left(k\right)=0.5\left(x^{\left(1\right)}\left(k\right)+x^{\left(1\right)}\left(k-1\right)\right),k=2,3,\cdots .n$.*

**Definition** **2.** *If $X^{\left(0\right)}$, $X^{\left(1\right)}$ and $Z^{\left(1\right)}$ are described as Definition 1, the following formula is obtained.*

(1) $$x^{\left(0\right)}\left(k\right)+az^{\left(1\right)}\left(k\right)=b{\left(z^{\left(1\right)}\left(k\right)\right)}^{r}$$

The above formula is the basic form of the GM(1,1) power model, and

(2) $$\frac{dx^{\left(1\right)}}{dt}+ax^{\left(1\right)}=b{\left(x^{\left(1\right)}\right)}^{r}$$

is the whitening equation of the GM(1,1) power model.

**Theorem** **1.** *If $X^{\left(0\right)}$, $X^{\left(1\right)}$ and $Z^{\left(1\right)}$ are shown in Definition 1, parameter list $\hat{a}={\left[a,b\right]}^{T}$ can be calculated by using the least squares method and shown as follows:*

(3) $$\hat{a}={\left[a,b\right]}^{T}={\left[B^{T}B\right]}^{-1}B^{T}Y$$

*where*

(4) $$B=\left[\begin{array}{cc} -z^{\left(1\right)}\left(2\right) & {\left[z^{\left(1\right)}\left(2\right)\right]}^{r}\\ -z^{\left(1\right)}\left(3\right) & {\left[z^{\left(1\right)}\left(3\right)\right]}^{r}\\ \vdots & \vdots \\ -z^{\left(1\right)}\left(n\right) & {\left[z^{\left(1\right)}\left(n\right)\right]}^{r} \end{array}\right],Y=\left[\begin{array}{c} x^{\left(0\right)}\left(2\right)\\ x^{\left(0\right)}\left(3\right)\\ \vdots \\ x^{\left(0\right)}\left(n\right) \end{array}\right]$$

**Definition** **3.** *Particularly, when r=2, then*

(5) $$x^{\left(0\right)}\left(k\right)+az^{\left(1\right)}\left(k\right)=b{\left(z^{\left(1\right)}\left(k\right)\right)}^{2}$$

*is the basic form of the grey Verhulst model, and*

(6) $$\frac{dx^{\left(1\right)}}{dt}+ax^{\left(1\right)}=b{\left(x^{\left(1\right)}\right)}^{2}$$

*is the whitening equation of the grey Verhulst model.*

**Theorem** **2.** *The solution to the whitening equation of the grey Verhulst model is shown as follows:*

(7) $${\hat{x}}^{\left(1\right)}\left(t\right)=\frac{ax^{\left(1\right)}\left(0\right)}{bx^{\left(1\right)}\left(0\right)+\left(a-bx^{\left(1\right)}\left(0\right)\right)e^{at}}$$

By substituting the initial value ${\hat{x}}^{\left(1\right)}\left(1\right)=x^{\left(1\right)}\left(1\right)$ into the above formula, the corresponding time response formula of the grey Verhulst model is obtained as:

(8) $${\hat{x}}^{\left(1\right)}\left(k+1\right)=\frac{ax^{\left(1\right)}\left(1\right)}{bx^{\left(1\right)}\left(1\right)+\left(a-bx^{\left(1\right)}\left(1\right)\right)e^{ak}}$$

Finally, it needs to be reverted to get the predicted value of the original sequence $x^{\left(0\right)}\left(k\right)$.

(9) $$x^{\left(0\right)}\left(k\right)=x^{\left(1\right)}\left(k\right)-x^{\left(1\right)}\left(k-1\right)$$

### 3.2. Derivation of Derived Form of the Grey Verhulst Model

In the modeling process of the traditional grey Verhulst model, a difference equation was firstly built and then converted into a differential equation, namely the whitening equation. Moreover, the whitening time response function was derived through integral operation, and the prediction and simulation were finally conducted. The transformation process inevitably led to the inherent deviation of the grey Verhulst model (see the work of Wang et al. [30]), so this study obtained a derived model of the grey Verhulst model by referring to the traditional GM(1,1) derived model proposed by Deng [44] and the method to derive the GM(1,1) power model by Wang [45]. This derived model did not need further prediction by virtue of the whitening response formula, which was a function that the traditional gray Verhulst model did not possess.

In order to facilitate the deduction of the derived models of the grey Verhulst model, the variable of the traditional GM(1,1) model is recorded as y and development coefficient is recorded as $a^{\prime }$. Moreover, the grey action quantity is expressed as $b^{\prime }$. The derived model, namely the GM(1,1,$y^{\left(1\right)}$) model, is defined as follows:

(10) $$y^{\left(0\right)}(k)=\beta^{\prime }-\alpha^{\prime }y^{\left(1\right)}(k-1)$$

where $\alpha^{\prime }=\frac{a^{\prime }}{1+0.5a^{\prime }}$ and $\beta^{\prime }=\frac{b^{\prime }}{1+0.5a^{\prime }}$.

**Theorem** **3.** *According to this derived model, the derived model of the grey Verhulst model can be obtained as follows:*

(11) $$x^{\left(1\right)}(k)=\frac{1}{\beta +(1-\alpha ){\left(x^{\left(1\right)}(k-1)\right)}^{-1}}$$

*where $\alpha =\frac{-a}{1-0.5a}$ and $\beta =\frac{-b}{1-0.5a}$.*

**Proof.**  Based on the derived $y^{\left(1\right)}$-type GM(1,1,$y^{\left(1\right)}$) model, the following formula can be derived. □

(12) $$\begin{array}{c} y^{\left(0\right)}(2)=\beta^{\prime }-\alpha^{\prime }y^{\left(1\right)}(1)\\ y^{\left(0\right)}(3)=\beta^{\prime }-\alpha^{\prime }y^{\left(1\right)}(2)\\ \vdots \\ y^{\left(0\right)}(k)=\beta^{\prime }-\alpha^{\prime }y^{\left(1\right)}(k-1) \end{array}$$

By adding the above k−1 formulas up,

(13) $$\sum_{i=2}^{k}y^{\left(0\right)}(i)=(k-1)\beta^{\prime }-\alpha^{\prime }\sum_{j=1}^{n-1}y^{\left(1\right)}(j)$$

By simultaneously adding $y^{\left(0\right)}(1)$ on both sides of the formula, the following formula is obtained:

(14) $$y^{\left(1\right)}(k)=(k-1)\beta^{\prime }+y^{\left(0\right)}(1)-\alpha^{\prime }\sum_{j=1}^{n-1}y^{\left(1\right)}(j)$$

By substituting $y^{\left(1\right)}(k)=\frac{1}{x^{\left(1\right)}(k)}$ into the above formula, the following equation can be obtained:

(15) $${\left(x^{\left(1\right)}(k)\right)}^{-1}=(k-1)\beta^{\prime }+{\left(x^{\left(0\right)}(1)\right)}^{-1}-\alpha^{\prime }{\sum_{j=1}^{n-1}\left(x^{\left(1\right)}(j)\right)}^{-1}$$

When k=2,

(16) $$\begin{array}{cc} {\left(x^{\left(1\right)}(2)\right)}^{-1} & =\beta^{\prime }+{\left(x^{\left(0\right)}(1)\right)}^{-1}-\alpha^{\prime }{\left(x^{\left(1\right)}(j)\right)}^{-1}\\ & =\beta^{\prime }+(1-\alpha^{\prime }){\left(x^{\left(0\right)}(1)\right)}^{-1} \end{array}$$

When k=3,4,…,n,

(17) $${\left(x^{\left(1\right)}(k-1)\right)}^{-1}=(k-2)\beta^{\prime }+{\left(x^{\left(0\right)}(1)\right)}^{-1}-\alpha^{\prime }{\sum_{j=1}^{k-2}\left(x^{\left(1\right)}(j)\right)}^{-1}$$

Therefore,

(18) $$\begin{array}{cc} {\left(x^{\left(1\right)}(k)\right)}^{-1} & =(k-1)\beta^{\prime }+{\left(x^{\left(0\right)}(1)\right)}^{-1}-\alpha^{\prime }{\sum_{j=1}^{n-1}\left(x^{\left(1\right)}(j)\right)}^{-1}\\ & =\beta^{\prime }+(k-2)\beta^{\prime }+{\left(x^{\left(0\right)}(1)\right)}^{-1}-\alpha^{\prime }{\sum_{j=1}^{k-2}\left(x^{\left(1\right)}(j)\right)}^{-1}-\alpha^{\prime }{\left(x^{\left(1\right)}(k-1)\right)}^{-1}\\ & =\beta^{\prime }+{\left(x^{\left(1\right)}(k-1)\right)}^{-1}-\alpha^{\prime }{\left(x^{\left(1\right)}(k-1)\right)}^{-1}\\ & =\beta^{\prime }+(1-\alpha^{\prime }){\left(x^{\left(1\right)}(k-1)\right)}^{-1} \end{array}$$

By substituting $\alpha^{\prime }=\frac{-a}{1-0.5a},\beta^{\prime }=\frac{-b}{1-0.5a}$ into the above formula, the following formula is obtained:

(19) $$\begin{array}{cc} {\left(x^{\left(1\right)}(k)\right)}^{-1} & =\beta^{\prime }+(1-\alpha^{\prime }){\left(x^{\left(1\right)}(k-1)\right)}^{-1}\\ & =\frac{-b}{1-0.5a}+(1+\frac{a}{1-0.5a}){\left(x^{\left(1\right)}(k-1)\right)}^{-1} \end{array}$$

that is, the derived model of the grey Verhulst model.

(20) $$x^{\left(1\right)}(k)=\frac{1}{\beta +(1-\alpha ){\left(x^{\left(1\right)}(k-1)\right)}^{-1}}$$

where $\alpha =\frac{-a}{1-0.5a}$ and $\beta =\frac{-b}{1-0.5a}$.

**Figure 3:**
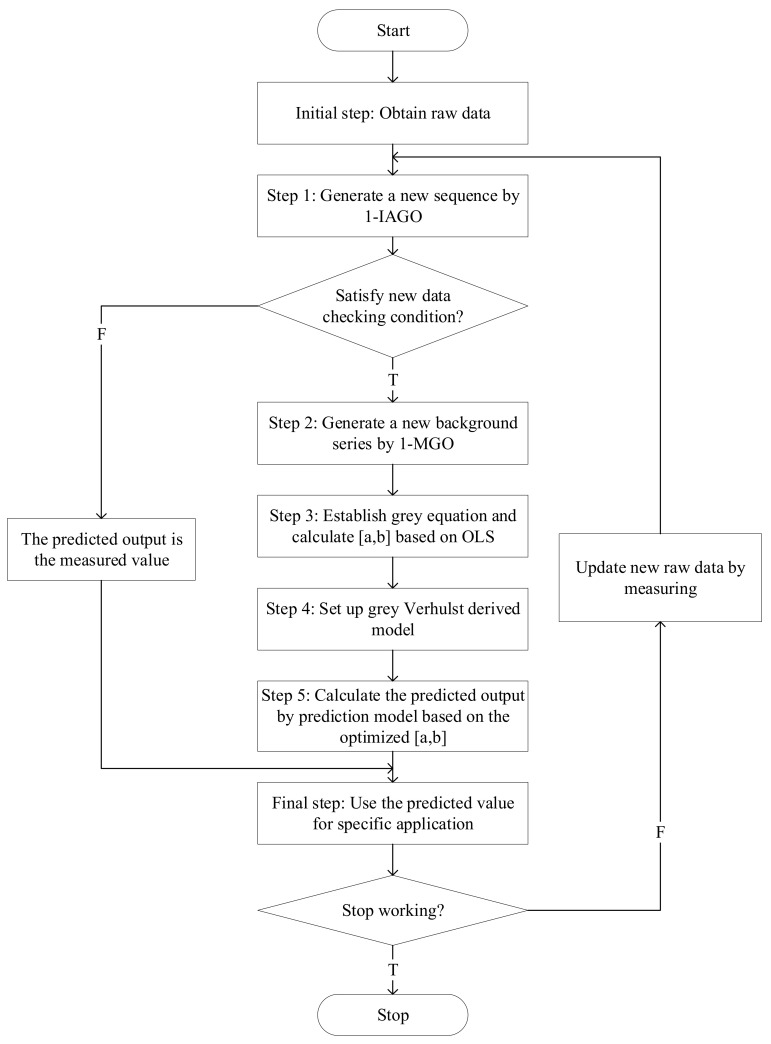
Flow chart of the derived grey Verhulst model.

The flow chart of the derived grey Verhulst model is shown in Figure 3.

### 3.3. Grey Verhulst Models with a Rolling Mechanism

**Figure 4:**
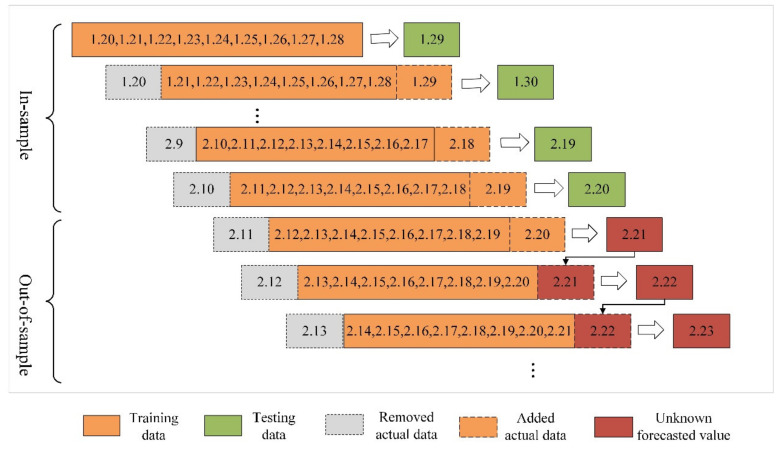
The rolling mechanism of the rolling grey Verhulst models.

When using the grey Verhulst model for modeling, the data before the real moment t=n are adopted. However, as time goes on, the development of any real socioeconomic system is accompanied by the constant access of some random disturbance factors, which affects the development of the system. A rolling mechanism can dynamically update the initial value of the data sequence and consider disturbance factors of the system, which has been proved to be able to greatly improve prediction accuracy [46]. Therefore, this study introduced a rolling mechanism into the grey Verhulst model and its derived model in order to reduce the influence of uncertain disturbance factors on the grey system in the future. The modeling process is presented as follows:

The traditional grey Verhulst model and its derived model established by the original data sequence $X^{\left(0\right)}=\left(x^{\left(0\right)}\left(1\right),x^{\left(0\right)}\left(2\right),\cdots ,x^{\left(0\right)}\left(n\right)\right)$ are used to predict the next value $x^{\left(0\right)}\left(n+1\right)$. By supplementing the value into the original sequence and removing the earliest data point $x^{\left(0\right)}\left(1\right)$, a new sequence, $X^{\left(0\right)}=\left(x^{\left(0\right)}\left(2\right),x^{\left(0\right)}\left(3\right),\cdots ,x^{\left(0\right)}\left(n\right),x^{\left(0\right)}\left(n+1\right)\right)$, is formed. This sequence is taken as the original sequence used to build the model, and the above steps are repeated for prediction and supplementation one by one. The models established according to the above steps are the grey rolling Verhulst model and its derived model. The length of the rolling sequence is expressed as L. When L is 9, the rolling modeling process of the two models is shown in Figure 4. This model can make good use of new information and obtain more accurate predicted results.

In order to compare accuracy and verify effectiveness and reliability of the models, absolute percentage error (APE) and mean absolute percentage error (MAPE) were used to calculate errors [47]:

(21) $$APE=\left|\frac{e\left(i\right)}{x^{\left(0\right)}\left(i\right)}\right|\times 100\%$$

(22) $$MAPE=\frac{1}{n}\sum_{i=1}^{n}\left|\frac{e\left(i\right)}{x^{\left(0\right)}\left(i\right)}\right|\times 100\%$$

where $e\left(i\right)=x^{\left(0\right)}\left(i\right)-{\hat{x}}^{\left(0\right)}\left(i\right)$, in which $x^{\left(0\right)}\left(i\right)$ and ${\hat{x}}^{\left(0\right)}\left(i\right)$ indicate actual value and predicted value, respectively. The levels of the accuracy of MAPE are shown in Table 1.

## 4. Empirical Analysis

Relevant data of the numbers of confirmed cases, suspected cases, cured patients and deaths were acquired by referring the latest data released by National Health Commission (http://www.nhc.gov.cn/). By using the number of patients infected with COVID-19 in China from 20 January to 20 February as the original data, empirical modeling and analysis were performed. Firstly, the rolling grey Verhulst model and its derived model were established, and the lengths of the rolling sequences were 7, 8 and 9. In accordance with the length of the rolling sequence, the original data were classified into a training set and a testing set, and the prediction accuracies of basic model and the derived model were compared. Secondly, in view of different lengths of the rolling sequences, the model with the highest prediction accuracy was selected to predict the final value and inflection point of the number of confirmed cases.

### 4.1. Parameter Estimation

In accordance with the rolling mechanism described in Section 3.3, the rolling grey Verhulst model and its derived model were built. By replacing the earliest data with the latest ones, the prediction and supplementation were carried out successively. Due to different analytic formulas of the general model and derived model, parameter estimation results are identical. By using the least squares method to estimate parameters of the two models, the parameter lists $\hat{a}={\left[a,b\right]}^{T}$ are obtained when the rolling sequence lengths are 7, 8 and 9. The results are shown in Table 2.

Parameters of the rolling grey Verhulst model and its derived model were obtained with different rolling sequence lengths, and predicted values were calculated based on recurrence prediction formula. 

### 4.2. Comparison of Model Accuracy

By establishing the rolling grey Verhulst model and its derived model with rolling sequence lengths of 7, 8 and 9, data from 20 January to 20 February 2020 were predicted, and the training and testing sets were established based on the length of the rolling sequence. On the basis of ensuring that the models could accurately simulate the number of confirmed patients in the training set, three models with high prediction accuracy in the testing set were selected to predict the maximum value and inflection point for different rolling sequence lengths. The results of the training and testing sets are presented in Table 3 and Table 4.

As displayed in Table 3, the prediction errors of the six models in the training set are less than 10%, indicating that the six models can accurately simulate changes of the number of confirmed cases in China. By observing prediction performance of the six models in the testing set, it can be seen that their prediction accuracies are all less than 10%, suggesting that the models can accurately predict changes of the number of confirmed cases in the future. As demonstrated in Table 4, in the testing set, prediction accuracy of the grey Verhulst model with the rolling sequence length of 7 is higher than that of its derived model; the grey Verhulst model and its derived model have MAPE values of 3.30% and 3.83%, respectively. When the rolling sequence lengths are 8 and 9, the grey Verhulst models show lower prediction accuracy than their derived models. MAPEs of the grey Verhulst model its derived model are 4.72% and 3.13%, respectively, when the rolling sequence length is 8. MAPEs of the grey Verhulst model its derived model are 2.93% and 1.65%, respectively, when the rolling sequence length is 9. Therefore, the grey Verhulst model with the rolling sequence length of 7 and derived grey Verhulst models with rolling sequence lengths of 8 and 9 were selected to predict the final value and inflection point of the number of confirmed cases in China.

### 4.3. Prediction of Final Value and Inflection Point of the Cumulative Number of Confirmed Patients

As shown in test results of models in Section 4.2, the grey Verhulst model with the rolling sequence length of 7 and derived grey Verhulst models with the rolling sequence lengths of 8 and 9 were used to predict final value and inflection point of the number of confirmed cases in China. In order to ensure combination of the rolling mechanism and the derived grey Verhulst models, for out-of-sample rolling prediction, the data from 12 to 20 February, 13 to 20 February and 14 to 20 February were used to predict the latest data, which served as the numbers of newly confirmed cases in the next period. Then, by removing the data on 12, 13 and 14 February, new initial sequences were established to continue the rolling operation until the predicted result in the latest day no longer changes, allowing this result to be taken as the final value.

On 13 February, Hubei Provincial Health Commission announced on its official website that, to be consistent with the case diagnosis and classification issued by other provinces in China, the province would release the number of clinically diagnosed cases and include this number in the confirmed cases. Since then, due to the appearance of clinically diagnosed cases, the number of confirmed patients increased greatly every day. Therefore, this study predicted changes of the number of confirmed patients with and without consideration of clinically diagnosed cases. This research further calculated out-of-sample prediction accuracies on 21 and 22 February to verify the predictive ability of the models. The results of final predicted values are demonstrated in Table 5. It was found that the three models could accurately predict out-of-sample data; the specific data are presented in Table 6.

**Figure 5:**
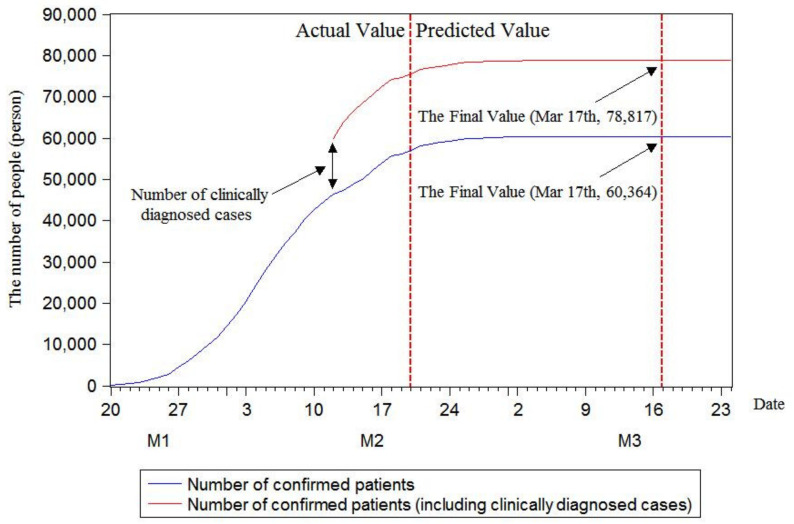
Model prediction with rolling sequence length of 7.

**Figure 6:**
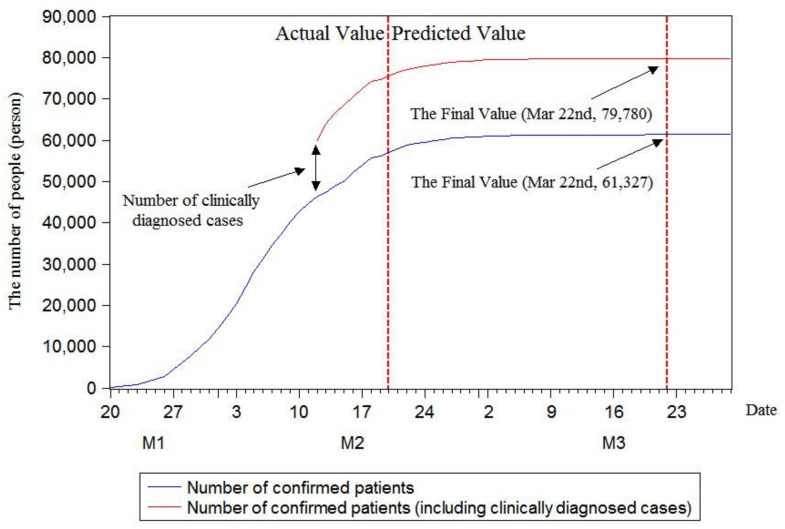
Model prediction with rolling sequence length of 8.

**Figure 7:**
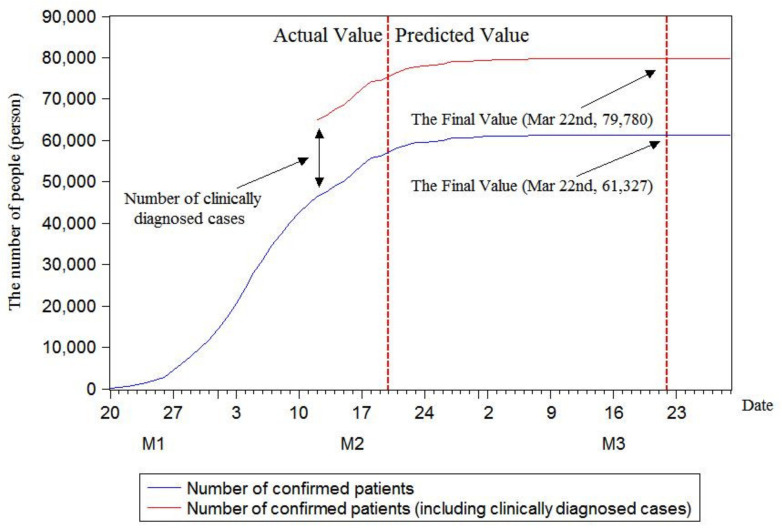
Model prediction with rolling sequence length of 9.

**Figure 8:**
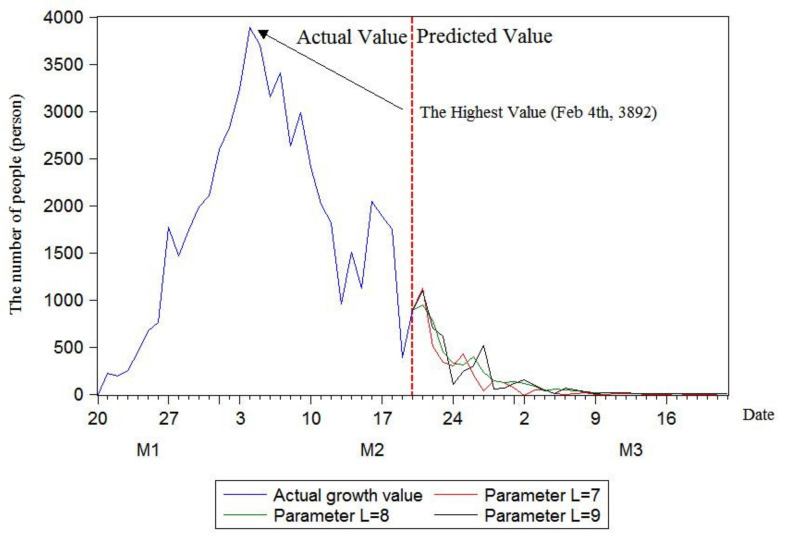
Changes in the daily growth of the number of confirmed patients in China.

As illustrated in Figure 5, Figure 6 and Figure 7, in the case of not considering clinically diagnosed cases, the maximum prediction values of the three models with rolling sequence lengths of 7, 8 and 9 are 60,364, 61,327 and 61,327, respectively. Under the condition of considering clinically diagnosed cases, the final prediction values are 78,817, 79,780 and 79,780 on 17, 22 and 22 March, respectively. By analyzing changes in the number of confirmed patients, it is found that the number of confirmed patients does show an S-shaped trend. Moreover, the current number of confirmed patients has been approximated to the final value, and the number of confirmed patients growing in a single day has decreased. In order to further calculate the inflection point, that is, the day with the maximum number of confirmed patients, this research plotted growth rate changes in single days using three models, as shown in Figure 8.

As shown in Figure 8, the inflection point had appeared on 4 February, with the number of confirmed patients of 3892. In addition, the growth in single days declines rapidly, and the predicted results of the three models are basically the same, which proves that the results are robust.

## 5. Conclusions

Based on a rolling mechanism, the rolling grey Verhulst model and its derived models for predicting the number of patients infected with COVID-19 in China were constructed by adding the latest data and removing the earliest data. Empirical modeling and analysis was conducted by using the number of infected cases from 20 January to 20 February. Firstly, in order to ensure stability of prediction results, the rolling sequence lengths of 7, 8 and 9 were selected for the models. The original data were classified into the training and testing sets to compare prediction accuracies of the basic model and the derived models. Secondly, considering the different rolling sequence lengths, the models with high precision accuracy were selected to predict the final value and inflection point of the number of confirmed patients. The results showed that the rolling grey Verhulst model and its derived models could accurately predict the changes in the number of confirmed patients in China. The prediction accuracy of the rolling grey Verhulst model with the rolling sequence length of 7 was higher than that of its derived model, while the prediction accuracies of the rolling grey Verhulst models with rolling sequence lengths of 8 and 9 were lower than those of the derived models. Therefore, this study used the rolling grey Verhulst models with high accuracy to predict the final number of confirmed patients and the date of reaching the final number.

By predicting the final number of confirmed patients in China using the rolling grey Verhulst model, the maximum predicted numbers by the three models with rolling sequence lengths of 7, 8 and 9 were 60,364, 61,327 and 61,327, respectively, when clinically diagnosed cases were not considered. If clinically diagnosed cases were considered, the final predicted values were 78,817, 79,780 and 79,780 on 17, 22 and 22 March, respectively. The prediction results of the inflection point using the rolling grey Verhulst model with rolling sequence lengths of 7, 8 and 9 showed that the inflection point appeared on 4 February when the number of confirmed patients increased by 3892.

## Figures and Tables

**Table 1:** The mean absolute percentage error (MAPE) criterion for model examination.

MAPE (%)	Predictive Ability	MAPE (%)	Predictive Ability
<10	Excellent	20–50	Reasonable
10–20	Good	>50	Incorrect

**Table 2:** The parameter values of the rolling grey Verhulst models.

Simulation→Forecast	Parameter *L* = 7		Simulation→Forecast	Parameter *L* = 8		Simulation→Forecast	Parameter *L* = 9	
	a	b		a	b		a	b
1.20–1.26→1.27	−0.4661	−5.84 × 10^−5^						
1.21–1.27→1.28	−0.2654	5.61 × 10^−5^	1.20–1.27→1.28	−0.2810	5.13 × 10^−5^			
1.22–1.28→1.29	−0.5013	−3.69 × 10^−5^	1.21–1.28→1.29	−0.4951	−3.56 × 10^−5^	1.20–1.28→1.29	−0.4988	−3.64 × 10^−5^
1.23–1.29→1.30	−0.5059	−3.72 × 10^−5^	1.22–1.29→1.30	−0.4967	−3.56 × 10^−5^	1.21–1.29→1.30	−0.4931	−3.50 × 10^−5^
1.24–1.30→1.31	−0.4658	−2.84 × 10^−5^	1.23–1.30→1.31	−0.4637	−2.81 × 10^−5^	1.22–1.30→1.31	−0.4594	−2.76 × 10^−5^
1.25–1.31→2.1	−0.4312	−2.27 × 10^−5^	1.24–1.31→2.1	−0.4324	−2.28 × 10^−5^	1.23–1.31→2.1	−0.4323	−2.28 × 10^−5^
1.26–2.1→2.2	−0.3728	−1.46 × 10^−5^	1.25–2.1→2.2	−0.3700	−1.44 × 10^−5^	1.24–2.1→2.2	−0.3740	−1.47 × 10^−5^
1.27–2.2→2.3	−0.2952	−7.58 × 10^−6^	1.26–2.2→2.3	−0.3370	−1.06 × 10^−5^	1.25–2.2→2.3	−0.3375	−1.07 × 10^−5^
1.28–2.3→2.4	−0.2666	−5.26 × 10^−6^	1.27–2.3→2.4	−0.2765	−5.86 × 10^−6^	1.26–2.3→2.4	−0.3058	−7.68 × 10^−6^
1.29–2.4→2.5	−0.2298	−2.74 × 10^−6^	1.28–2.4→2.5	−0.2420	−3.37 × 10^−6^	1.27–2.4→2.5	−0.2511	−3.84 × 10^−6^
1.30–2.5→2.6	−0.2408	−3.57 × 10^−6^	1.29–2.5→2.6	−0.2467	−3.83 × 10^−6^	1.28–2.5→2.6	−0.2532	−4.12 × 10^−6^
1.31–2.6→2.7	−0.2787	−5.51 × 10^−6^	1.30–2.6→2.7	−0.2693	−5.15 × 10^−6^	1.29–2.6→2.7	−0.2700	−5.18 × 10^−6^
2.1–2.7→2.8	−0.2763	−5.34 × 10^−6^	1.31–2.7→2.8	−0.2722	−5.20 × 10^−6^	1.30–2.7→2.8	−0.2659	−4.98 × 10^−6^
2.2–2.8→2.9	−0.3030	−6.31 × 10^−6^	2.1–2.8→2.9	−0.2886	−5.86 × 10^−6^	1.31–2.8→2.9	−0.2833	−5.69 × 10^−6^
2.3–2.9→2.10	−0.2880	−5.69 × 10^−6^	2.2–2.9→2.10	−0.2812	−5.49 × 10^−6^	2.1–2.9→2.10	−0.2738	−5.28 × 10^−6^
2.4–2.10→2.11	−0.2590	−4.86 × 10^−6^	2.3–2.10→2.11	−0.2779	−5.35 × 10^−6^	2.2–2.10→2.11	−0.2746	−5.26 × 10^−6^
2.5–2.11→2.12	−0.2424	−4.45 × 10^−6^	2.4–2.11→2.12	−0.2805	−5.44 × 10^−6^	2.3–2.11→2.12	−0.2773	−5.33 × 10^−6^
2.6–2.12→2.13	−0.2516	−4.67 × 10^−6^	2.5–2.12→2.13	−0.2418	−4.43 × 10^−6^	2.4–2.12→2.13	−0.2565	−4.79 × 10^−6^
2.7–2.13→2.14	−0.2646	−5.04 × 10^−6^	2.6–2.13→2.14	−0.2735	−5.25 × 10^−6^	2.5–2.13→2.14	−0.2594	−4.92 × 10^−6^
2.8–2.14→2.15	−0.2803	−5.33 × 10^−6^	2.7–2.14→2.15	−0.2446	−4.55 × 10^−6^	2.6–2.14→2.15	−0.2579	−4.84 × 10^−6^
2.9–2.15→2.16	−0.2375	−4.38 × 10^−6^	2.8–2.15→2.16	−0.2646	−4.96 × 10^−6^	2.7–2.15→2.16	−0.2390	−4.41 × 10^−6^
2.10–2.16→2.17	−0.0905	−1.20 × 10^−6^	2.9–2.16→2.17	−0.1483	−2.39 × 10^−6^	2.8–2.16→2.17	−0.1980	−3.42 × 10^−6^
2.11–2.17→2.18	0.0071	7.89 × 10^−7^	2.10–2.17→2.18	−0.0550	−4.43 × 10^−7^	2.9–2.17→2.18	−0.1077	−1.50 × 10^−6^
2.12–2.18→2.19	0.0405	1.40 × 10^−6^	2.11–2.18→2.19	−0.0099	4.36 × 10^−7^	2.10–2.18→2.19	−0.0514	−3.67 × 10^−7^
2.13–2.19→2.20	−0.1216	−1.80 × 10^−6^	2.12–2.19→2.20	−0.0685	−8.07 × 10^−7^	2.11–2.19→2.20	−0.0884	−1.18 × 10^−6^
2.14–2.20→2.21	−0.1662	−2.63 × 10^−6^	2.13–2.20→2.21	−0.1360	−2.08 × 10^−6^	2.12–2.20→2.21	−0.0874	−1.19 × 10^−6^

**Table 3:** APE values and MAPE values of the training set.

Date	Rolling Grey Verhulst Model			Rolling Grey Verhulst Derived Model		
	Parameter *L* = 7	Parameter *L* = 8	Parameter *L* = 9	Parameter *L* = 7	Parameter *L* = 8	Parameter *L* = 9
1.26	10.21			6.32		
1.27	14.34	38.25		4.89	7.94	
1.28	16.27	22.80	9.83	7.17	7.66	8.96
1.29	6.39	14.92	21.49	5.05	6.19	6.77
1.30	2.34	2.61	8.76	4.00	3.82	4.64
1.31	3.47	3.55	3.36	3.75	3.41	3.05
2.1	8.43	7.03	9.31	3.53	3.30	3.61
2.2	1.76	10.75	10.00	0.79	3.05	2.77
2.3	1.40	2.83	13.52	0.74	1.00	2.98
2.4	1.25	2.74	4.99	0.71	1.04	1.43
2.5	0.44	0.53	1.80	0.54	0.67	0.93
2.6	0.82	1.30	1.09	0.83	0.86	0.80
2.7	0.61	0.68	1.09	0.73	0.70	0.74
2.8	0.38	0.66	0.85	0.65	0.80	0.82
2.9	0.55	0.50	0.58	0.65	0.69	0.75
2.10	0.31	0.61	0.55	0.53	0.62	0.62
2.11	0.25	0.56	0.56	0.40	0.47	0.56
2.12	0.20	0.22	0.27	0.33	0.36	0.44
2.13	0.33	0.29	0.35	0.45	0.41	0.49
2.14	0.22	0.33	0.29	0.33	0.43	0.43
2.15	0.24	0.27	0.33	0.29	0.33	0.39
2.16	0.52	0.70	0.84	0.65	0.60	0.61
2.17	0.33	0.55	0.84	0.56	0.67	0.70
2.18	0.28	0.28	0.50	0.45	0.53	0.60
2.19	0.58	0.73	0.66	0.77	0.79	0.77
MAPE	2.88	4.74	3.99	1.80	1.93	1.91

**Table 4:** Predicted values and MAPE values of the testing set.

Date	Actual Value	Rolling Grey Verhulst Model						Rolling Grey Verhulst Derived Model					
		Parameter *L* = 7		Parameter *L* = 8		Parameter *L* = 9		Parameter *L* = 7		Parameter *L* = 8		Parameter *L* = 9	
		Forecasted Value	APE (%)	Forecasted Value	APE (%)	Forecasted Value	APE (%)	Forecasted Value	APE (%)	Forecasted Value	APE (%)	Forecasted Value	APE (%)
1.27	4535	3388	25.30					3670	19.08				
1.28	5999	5678	5.35	3135	47.74			8383	39.73	8250	37.53		
1.29	7736	8426	8.92	8784	13.54	8101	4.72	7727	0.11	7747	0.14	7735	0.02
1.30	9720	9597	1.27	9995	2.83	10,301	5.98	9370	3.60	9401	3.29	9413	3.16
1.31	11,821	11,424	3.36	11,491	2.79	11,857	0.31	11,485	2.84	11,493	2.77	11,510	2.63
2.1	14,413	13,438	6.77	13,377	7.19	13,358	7.32	13,652	5.28	13,647	5.32	13,647	5.31
2.2	17,238	15,874	7.92	15,948	7.49	15,621	9.38	16,691	3.17	16,706	3.08	16,684	3.22
2.3	20,471	19,853	3.02	18,557	9.35	18,501	9.62	20,121	1.71	19,845	3.06	19,841	3.08
2.4	24,363	23,555	3.32	23,246	4.58	21,456	11.93	23,812	2.26	23,735	2.58	23,499	3.55
2.5	28,060	28,234	0.62	27,806	0.91	27,234	2.94	28,535	1.69	28,418	1.27	28,326	0.95
2.6	31,211	32,094	2.83	31,910	2.24	31,568	1.14	32,083	2.80	32,028	2.62	31,963	2.41
2.7	34,621	34,499	0.35	34,677	0.16	34,642	0.06	34,436	0.53	34,514	0.31	34,507	0.33
2.8	37,251	37,648	1.07	37,705	1.22	37,856	1.63	37,645	1.06	37,679	1.15	37,735	1.30
2.9	40,235	39,567	1.66	39,725	1.27	39,807	1.06	39,592	1.60	39,694	1.34	39,735	1.24
2.10	42,638	42,334	0.71	42,399	0.56	42,497	0.33	42,431	0.49	42,479	0.37	42,537	0.24
2.11	44,653	44,631	0.05	44,548	0.23	44,582	0.16	44,684	0.07	44,542	0.25	44,570	0.19
2.12	46,472	46,442	0.06	46,004	1.01	46,148	0.70	46,460	0.03	46,358	0.24	46,246	0.49
2.13	47,424	47,920	1.05	47,981	1.17	47,862	0.92	47,950	1.11	48,000	1.22	47,915	1.03
2.14	48,927	48,538	0.80	48,493	0.89	48,577	0.71	48,513	0.85	48,479	0.92	48,542	0.79
2.15	50,047	49,725	0.64	49,891	0.31	49,812	0.47	49,783	0.53	49,912	0.27	49,855	0.38
2.16	52,095	50,827	2.43	50,711	2.66	50,844	2.40	50,868	2.36	50,782	2.52	50,877	2.34
2.17	53,983	53,503	0.89	53,216	1.42	52,907	1.99	53,517	0.86	53,271	1.32	53,042	1.74
2.18	55,732	55,947	0.39	55,650	0.15	55,346	0.69	55,977	0.44	55,668	0.11	55,390	0.61
2.19	56,123	57,985	3.32	57,682	2.78	57,447	2.36	57,950	3.25	57,695	2.80	57,467	2.40
2.20	57,012	57,274	0.46	57,426	0.73	57,355	0.60	57,250	0.42	57,410	0.70	57,339	0.57
MAPE			3.30		4.72		2.93		3.83		3.13		1.65

**Table 5:** The distributions of predicted values.

Date	Parameter *L* = 7		Parameter *L* = 8		Parameter *L* = 9	
	Predicted Value	Predicted Value (Including Clinically Diagnosed Cases)	Predicted Value	Predicted Value (Including Clinically Diagnosed Cases)	Predicted Value	Predicted Value (Including Clinically Diagnosed Cases)
2.21	58,136	76,589	57,959	76,412	58,108	76,561
2.22	58,638	77,091	58,741	77,194	58,811	77,264
2.23	58,973	77,426	59,183	77,636	59,423	77,876
2.24	59,276	77,729	59,514	77,967	59,530	77,983
2.25	59,706	78,159	59,820	78,273	59,769	78,222
2.26	59,904	78,357	60,218	78,671	60,066	78,519
2.27	59,938	78,391	60,447	78,900	60,577	79,030
2.28	60,085	78,538	60,588	79,041	60,632	79,085
2.29	60,207	78,660	60,710	79,163	60,696	79,149
3.1	60,273	78,726	60,847	79,300	60,806	79,259
3.2	60,261	78,714	60,962	79,415	60,962	79,415
3.3	60,302	78,755	61,041	79,494	61,059	79,512
3.4	60,348	78,801	61,079	79,532	61,104	79,557
3.5	60,350	78,803	61,129	79,582	61,108	79,561
3.6	60,346	78,799	61,177	79,630	61,170	79,623
3.7	60,355	78,808	61,213	79,666	61,217	79,670
3.8	60,367	78,820	61,233	79,686	61,242	79,695
3.9	60,365	78,818	61,249	79,702	61,249	79,702
3.10	60,360	78,813	61,266	79,719	61,262	79,715
3.11	60,362	78,815	61,283	79,736	61,280	79,733
3.12	60,364	78,817	61,293	79,746	61,297	79,750
3.13	60,365	78,818	61,299	79,752	61,300	79,753
3.14	60,364	78,817	61,305	79,758	61,303	79,756
3.15	60,363	78,816	61,311	79,764	61,309	79,762
3.16	60,362	78,815	61,315	79,768	61,316	79,769
3.17	60,364	78,817	61,318	79,771	61,319	79,772
3.18			61,320	79,773	61,321	79,774
3.19			61,322	79,775	61,322	79,775
3.20			61,324	79,777	61,324	79,777
3.21			61,326	79,779	61,326	79,779
3.22			61,327	79,780	61,327	79,780

**Table 6:** Out-of-sample prediction accuracy.

Date	Actual Value	Parameter *L* = 7		Parameter *L* = 8		Parameter *L* = 9	
		Predicted Value	APE (%)	Predicted Value	APE (%)	Predicted Value	APE (%)
2.21	76,288	76,589	0.39	76,412	0.16	76,561	0.36
2.22	76,936	77,091	0.20	77,194	0.34	77,264	0.43
MAPE			0.30		0.25		0.39
